# Astragaloside IV improves MASLD via GCN5-LXRα/SREBP1c signaling pathway-mediated hepatic de novo lipogenesis

**DOI:** 10.1186/s13020-026-01491-0

**Published:** 2026-09-28

**Authors:** Hui-Mei Liang, Yin-Yue Xu, Kun Miao, Hai-Tao Xiao, Yong-Dan Wang, Jing Jin, He Wu, Bao-Yi Chen, Li-Jun Xu, Jiang-Ping Zhu, Li-Na Lai, Zu-Guo Zheng

**Affiliations:** 1 Department of Ultrasound, The First Affiliated Hospital, Fujian Medical University, Fuzhou, 350005 China; 2 State Key Laboratory of Natural Medicines, Department of Pharmacognosy, School of Traditional Chinese Pharmacy, China Pharmaceutical University, No. 639 Long Mian Avenue, Nanjing, 211198 Jiangsu China; 3 Department of Hand Surgery, Fuzhou Second General Hospital, Fuzhou, 350007 Fujian China; 4 School of Pharmacy, Changzhi Medical College, 161 Jiefang East Street, Changzhi, 046099 Shanxi China; 5 Xizang University of Tibetan Medicine, Lhasa, 850000 Tibet China; 6 College of Computer Science, Sichuan University, Chengdu, 610065 China

**Keywords:** Astragaloside IV, GCN5, LXRα, SREBP1c, MASLD, De novo lipogenesis

## Abstract

**Background:**

Astragaloside IV (AS-IV), a bioactive saponin from Astragalus membranaceus, shows promise for treating metabolic dysfunction-associated steatotic liver disease (MASLD), though its mechanisms remain unclear.

**Purpose:**

This study aimed to elucidate how AS-IV alleviates hepatic steatosis, specifically investigating its epigenetic regulation of lipogenesis.

**Methods:**

Using high-fat diet-fed mice and lipids-loaded hepatocytes, we assessed AS-IV's effects through SREBP-1c overexpression, histone acetylation analysis, and GCN5 rescue experiments.

**Results:**

AS-IV significantly reduced hepatic steatosis by suppressing SREBP-1c-mediated de novo lipogenesis. Mechanistically, it inhibited histone acetyltransferase GCN5, decreasing H3K9/H3K14 acetylation at the SREBP-1c promoter and attenuating LXRα-driven transcription. GCN5 overexpression reversed AS-IV's suppression of SREBP-1c and lipid accumulation.

**Conclusion:**

AS-IV uniquely downregulates GCN5 expression at the transcriptional level, exerting epigenetic regulation to disrupts lipogenic programming, This represents a novel multi-target approach against MASLD that connects natural product pharmacology with epigenetic modulation.

**Supplementary Information:**

The online version contains supplementary material available at https://doi.org/10.1186/s13020-026-01491-0.

## Introduction

Metabolic dysfunction-associated steatotic liver disease (MASLD) is identified by hepatic steatosis without excessive alcohol consumption, is associated with metabolic dysregulation such as obesity, insulin resistance, or type 2 diabetes [1, 2]. MASLD impacts about 25% of the world's population. Its prevalence is rising rapidly, particularly in Asia due to urbanization and dietary shifts [3, 4]. Currently, no pharmacotherapies are approved for MASLD; vitamin E and pioglitazone have limited efficacy and safety concerns [5]. Lifestyle interventions remain cornerstone therapies but suffer from poor long-term adherence. In 2024, the FDA approved Resmetirom, an oral selective thyroid hormone receptor (THR)-β agonist, for treating MASH with liver fibrosis [6]. This approval signified the introduction of the sole new drug authorized for MASH treatment [5, 6]. However, identifying novel agents targeting MASLD pathogenesis is urgently needed.

Hepatic lipid accumulation, characteristic of MASLD, results from an imbalance in lipid uptake, synthesis, and export. In MASLD, de novo lipogenesis (DNL) contributes up to 30% of hepatic triglycerides through increased expression of acetyl-CoA carboxylase (ACC) and fatty acid synthase (FAS) [7]. Insulin resistance and hyperinsulinemia further exacerbate DNL by activating lipogenic transcription factors. Excess free fatty acids promote lipotoxicity, mitochondrial dysfunction, and oxidative stress, perpetuating hepatic injury and inflammation [8]. Targeting DNL is a therapeutic priority, but current inhibitors face specificity and tolerability issues, highlighting the need for alternative strategies.

Sterol regulatory element-binding protein 1c (SREBP-1c) is a key transcription factor driving DNL by activating ACC, FAS, and SCD1 [9]. In MASLD, SREBP-1c is hyperactivated through insulin signaling and liver X receptor α (LXRα)-dependent pathways, leading to lipid accumulation [10]. Clinical studies demonstrate elevated SREBP-1c precursors and mature forms in MASLD patients, correlating with disease severity [11]. Inhibiting SREBP-1c, either genetically or pharmacologically, reduces hepatic steatosis in preclinical models, highlighting its therapeutic potential [7]. However, direct targeting of SREBP‑1c is challenging, prompting interest in upstream epigenetic regulators. General control non-depressible 5 (GCN5), a histone acetyltransferase, acetylates H3K9/H3K14 and enhances LXRα‑mediated SREBP‑1c transcription and DNL [12], its pharmacological inhibition ameliorates hepatic steatosis, suggesting GCN5 as an epigenetic checkpoint [12]. Yet, the interplay among GCN5, LXRα and SREBP‑1c in MASLD remains underexplored. Notably, natural compounds like resveratrol exert anti-steatotic effects via GCN5 suppression, underscoring the therapeutic viability of epigenetic modifiers [13, 14].

However, the interplay between GCN5, LXRα, and SREBP-1c in MASLD remains underexplored, offering a novel avenue for intervention.

Astragali Radix, recognized as a fundamental herb for tonifying Qi in traditional Chinese medicine, is historically documented to restore deficiency of the five viscera. As noted in the TCM Classical Texts: Ben Cao Bu Yi, "when processed with honey, it fortifies the middle energizer, augments primordial Qi, warms the triple energizer, and strengthens the spleen and stomach". Astragaloside IV (AS-IV), a tetracyclic triterpenoid saponin, is one of the major active constituents of Astragalus and is commonly used as a key indicator to evaluate the quality of Astragalus medicinal materials [15–17]. Studies have shown that AS-IV exerts hepatoprotective effects by modulating signaling pathways such as TLR4/NF-κB, thereby reducing serum levels of IL-6, IL-8, and TNF-α while increasing IL-10 levels. Moreover, AS-IV significantly decreases transaminase activity, ameliorates lipid accumulation and oxidative injury, and consequently slows the progression of hepatic disorders [18]. Building on these insights, our study investigates Astragaloside IV (AS-IV), a bioactive saponin from *Astragalus membranaceus*, demonstrating anti-steatotic effects in preclinical models. Notably, the transcription factor SREBP-1c is primarily activated by liver X receptor α (LXRα), which itself relies on epigenetic co‑factors such as GCN5 for full transcriptional output. We reveal that AS-IV suppresses DNL by epigenetically inhibiting SREBP-1c via GCN5-dependent histone deacetylation, a mechanism distinct from existing therapies. This work bridges the gap between natural product pharmacology and epigenetic regulation, offering a promising strategy for MASLD treatment.

## Materials and methods

### Materials and reagents

Bovine Serum Albumin (BSA) (B2064), compactin (1443216), Oil red O (O0625), oleic acid (OA) (O1008), palmitic acid (PA) (P5585), puromycin (P7255), and Triton X-100 (T8787) were purchased from Sigma-Aldrich. Nile Red (SS1956) was purchased from BIOFUNT. Lipofectamine 3000 (L3000008) and BODIPY^493/503^ (D3922) were purchased from Thermo Fisher Scientific. The Simple ChIP Plus Sonication Chromatin IP Kit (56383S) was obtained from Cell Signaling Technology. Protease inhibitors (04693124001) and Phosphatase inhibitors (4906845001) were obtained from Roche. T0901317 (HY-10626) and GW3965 (HY-10627) were purchased from MedChemExpress. High-fat diets (D12492) were obtained from Research Diets. Normal diets (1010039) were purchased from Jiangsu Synergetic Biology Co., Ltd. Assay kits for TG (A110-1–1), TC (A111-1–1), HDL-c (A112-1–1), and LDL-c (A113-1–1) were procured from Nanjing Jiancheng Bioengineering Institute. RNA-easy isolated reagent (R701), HiScript Reverse Transcriptase kit (R123-01), Dual Luciferase Reporter Assay Kit (DL101-01), and qPCR SYBR Green Master Mix (Q111-02) were purchased from Vazyme. Protein A/G agarose bead (sc-2003) was purchased from Santa Cruz Biotech.

### Primary antibodies

Antibodies, Anti-Flag (ab205606) and Anti-SREBP1 (ab3259), were obtained from Abcam. The anti-GAPDH antibody (AF0006) was obtained from Beyotime. Anti-GCN5 (A2224) and anti-Myc (AE010) antibodies were purchased from Abclonal. Antibodies anti-H3K14ac (7627 T) and anti-H3K9ac (9649S) were obtained from Cell Signaling Technology.

### Plasmids

The plasmids used in this study were constructed as follows. The coding sequences (CDSs) of human GCN5 (also known as KAT2A, which belongs to the GCN5‑related N‑acetyltransferase (GNAT) superfamily, NCBI Reference Sequence: NM_021078.3), human SREBP1c (NCBI: NM_004176.5), human RXRα (NCBI: NM_002957.6), and human LXRα (NCBI: NM_005693.4) were amplified from the cDNA library of HL‑7702 cells by PCR and cloned into the pCMV3‑C‑Flag, pCMV3‑C‑HA, or pCMV3‑C‑Myc vector (Sino Biological Inc., Beijing, China) via standard restriction enzyme digestion and ligation. The resulting constructs were designated as pCMV3‑C‑Flag‑GCN5 (full‑length GCN5), pCMV3‑C‑Flag‑p‑SREBP1c (precursor form, encoding amino acids 1–1147), pCMV3‑C‑Flag‑m‑SREBP1c (mature nuclear form, encoding amino acids 1–490), pCMV3‑C‑HA‑RXRα, and pCMV3‑C‑Myc‑LXRα. The acetyltransferase‑deficient mutant GCN5 (E575Q) was generated by site‑directed mutagenesis using the wild‑type GCN5 construct as the template, with the glutamate at position 575 replaced by glutamine, and was confirmed by Sanger sequencing. All plasmid sequences were verified by full‑length sequencing (Sangon Biotech, Shanghai, China) prior to use. Primer sequences used for cloning and mutagenesis are available upon reasonable request.

### Cell culture

HL-7702 cells, obtained from Keygen Biotechnology, were used as a model for normal human hepatocytes, while HEK293T cells were purchased from the American Type Culture Collection. All cell lines were cultured at 37 °C and 5% CO_2_ in the indicated medium.

### Culture medium

Medium A consists of DMEM (Keygen, KGL1206) with 100 μ/ml penicillin, 100 μg/ml streptomycin sulfate, and 10% fetal bovine serum (Gibco, A5670701). A medium composed of an equal volume mixture of Ham’s F-12 K medium (Gibco, 21,127,030) and DMEM, supplemented with 5% LPDS (Kalen Biomedical, 880,100), 10 μM compactin (Sigma-Aldrich, 1,443,216), and 50 μM mevalonate (Aladdin, D304342).

### Mouse primary hepatocyte isolation

During all experiments, mice were anesthetized with 1.5% isoflurane in O_2_, followed by catheter insertion into the vena cava and portal vein incision. The livers were then perfused with oxygenated buffer A (1 × PBS, 5 mM EGTA) at 37 °C. The liver was perfused with oxygenated buffer B (1 × PBS, 1 mM CaCl_2_, collagenase type IV) at 37 °C, then transferred to a Petri dish containing buffer C (1 × PBS, 2 mM CaCl_2_, 0.6% BSA) and disaggregated with forceps. Following digestion with sterile gauze, liver perfusions were centrifuged at 48*g* for 5 min. Primary hepatocytes were subsequently isolated from mice using sterile gauze. Hepatocyte pellets were resuspended in Medium B, and the supernatant was discarded following three washes. Hepatocytes were cultured on collagen-coated plates, and cell viability was assessed using the trypan blue exclusion test. A cell viability exceeding 70% was deemed sufficient to proceed with the research.

### Viability assay

The viability of HL-7702 cells was assessed via the MTT assay. In summary, 10,000 cells were plated per well in 96-well plates. Cells were exposed to the specified concentration of CPTH2 for 24 h following an initial 24 h period. MTT (10 μl, 5 mg/ml) was introduced to each well and incubated at 37 ℃ for 4 h, followed by the addition of formazan (100 μl) and further incubation at 37 ℃ for 3–4 h. Absorbance was measured at 570 nm using a BMG POLARstar Omega microplate reader.

### BODIPY^493/503^ and Nile Red staining

Cells were fixed with 4% paraformaldehyde and then stained with 1 μM Nile Red or 5 μM BODIPY^493/503^ for 30 min at room temperature in the dark. The sample was rinsed three times with PBS to remove excess dye. Intracellular lipid droplets were imaged for fluorescence using either a laser confocal microscope (Olympus FV3000) or an inverted fluorescence microscope (Nikon Ts2R). Intracellular neutral lipids were quantified using ImageJ (Version 1.5a).

### Western blotting

Cells were harvested and lysed using a cell lysis buffer containing SDS, loading, protease inhibitor, and phosphatase inhibitor. Cell lysates were subjected to 10 min of heating at 95 °C, followed by separation using 8–12% SDS-PAGE and transfer onto nitrocellulose membranes. Membranes were blocked with 5% non-fat milk for 1 h at room temperature, then incubated with primary antibodies overnight at 4 °C, followed by a 1-h incubation with HRP-conjugated secondary antibodies at room temperature. The immunoblots were visualized by chemiluminescence and imaged using a Tanon 5200 imaging system (Tanon, China). Protein quantification was performed using ImageJ (Version 1.5a).

### Lipid analysis in cellular, hepatic, and serum samples.

A portion of cells were kept for protein content determination (BCA assay) and the remaining cells were subjected to lipid extraction. Cells’ total cholesterol (TC) and triacylglycerol (TG) were extracted using 1 ml of a 2:1 chloroform–methanol mixture for 3 h at room temperature. After adding 500 μl NaCl (0.1 M), the samples were vortexed for 1 min and centrifuged for phase separation. The lipid-containing nonpolar layer was collected and dried using a gentle nitrogen stream. The samples were resuspended in 50 μl of ethanol with 1% Triton X-100 (Sigma-Aldrich, T9284). TG and TC levels were assessed using respective assay kits as per the manufacturer's guidelines and normalized to protein concentration.

To quantify intrahepatic TC and TG, 50 mg of liver tissue was homogenized in 0.5 ml of PBS. A segment of liver homogenates was set aside for protein content analysis using the BCA assay, while 0.4 ml of the homogenates were combined with 1.6 ml of a chloroform and methanol mixture (2:1, v/v) for lipid extraction as previously described.

Serum concentrations of TC, TG, HDL-c, and LDL-c were measured using kits following the manufacturer's instructions.

### Measurement of de novo fatty acid synthesis

*KAT2A* knockout and wild-type HL-7702 cells were cultured in medium B with indicated treatment for 16 h. The rate of de novo fatty acid synthesis was evaluated by incubating the cells with 12 µCi of [^14^C] acetate per 60 mm dish for 2 additional hours. Post-incubation, cells were washed, lysed in 0.1 N NaOH, and subjected to autoclaving for alkaline saponification.Then the nonpolar lipids (cholesterol) were extracted in petroleum ether and dried under a nitrogen stream. Polar lipids were extracted in petroleum ether following acidification with concentrated HCl and subsequently dried under a nitrogen stream. Subsequently, 5 ml of scintillation cocktail was added to the sample to measure the DPM value.

### Reporter gene assay

Promoter-reporter constructs such as SREBP-1c-Luc, SREBP-1c (mtLXRE)-Luc, ABCA1-Luc, and ABCA1 (mtLXRE)-Luc were created via a PCR-cloning strategy. HL-7702 cells were seeds into 96-well plates and treated as described. HL-7702 cells underwent transfection with specified luciferase reporter plasmids and received the described treatment. Cells were lysed for 30 min at room temperature using the reporter gene cell lysis buffer (Beyotime, RG0036). Cell lysates of each well were transferred into a white 96-well plate. Luciferase substrate (Vazyme, DL101-01) was added to cell lysates under light-protected conditions, and luminescence intensity was promptly measured using a BMG POLARstar Omega microplate reader. The luminescence intensity was normalized to the protein concentration of each well.

### qRT-PCR

RNAs were extracted using the RNA-easy isolation reagent. cDNA was synthesized using the HiScript Reverse Transcriptase kit. Quantitative real-time PCR (qRT-PCR) was conducted using SYBR Green Master Mix on a LightCycler 96 system (Roche). Gene expression levels were determined using the △△CT method. Refer to Table 1 for the primer sequences utilized.

**Table 1:** Primers for RT-qPCR

Gene	Forward	Reverse
*Mus musculus*		
*Kat2a*	AATTCTCCCATCTGGGAGTCAGG	ATGGAAGGACTGAAGCTGGGTAC
*Kat2b*	CCGTGTCATTGGTGGTATCTGTT	AGGAAGTTGAGGATCTCGTGCTT
*β-actin*	GGCTGTATTCCCCTCCATCG	CCAGTTGGTAACAATGCCATG
*Srebf1*	GGAGCCATGGATTGCACATT	GGCCCGGGAAGTCACTGT
*Acc*	GCCCCATATGATCCTCGGTG	ATTCCCCCTAACCTGGCTCT
*Fasn*	CTGACTCGGCTACTGACACG	AATGGGGTGCACAAGGAACA
*Scd1*	GGGTGCCGTGGGCGA	GGAACTCAGAAGCCCAAAGC
*Scd2*	GCATTTGGGAGCCTTGTACG	AGCCGTGCCTTGTATGTTCTG
*Fads2*	GGCCACTTAAAGGGTGCCTC	GGCTCTTTATGTCCGGGTCC
*Mcad*	AAACATGGGCCAGCGATGCTCT	AGGGCATACTTCGTGGCTTCGT
*Pdk4*	TTCACACCTTCACCACATGC	AAAGGGCGGTTTTCTTGAT
*Fatp1*	CGCTTTCTGCGTATCGTCTG	GATGCACGGGATCGTGTCT
*Ldlr*	TGACTCAGACGAACAAGGCTG	ATCTAGGCAATCTCGGTCTCC
*Cd36*	ATGGGCTGTGATCGGAACTG	GTCTTCCCAATAAGCATGTCTCC
*Apob*	AAGCACCTCCGAAAGTACGTG	CTCCAGCTCTACCTTACAGTTGA
*Apoe*	CTGACAGGATGCCTAGCCG	CGCAGGTAATCCCAGAAGC
*Fatp4*	TGTGGTGCACAGCAGGTATT	AGTCATGCCGTGGAGTAAGC
*Ucp1*	ATGGTTCAGAAGCCGAGCAA	CCACCCACGAGACATAGAGC
*Mttp*	CTCTTGGCAGTGCTTTTTCTCT	GAGCTTGTATAGCCGCTCATT
*Pparα*	AGAGCCCCATCTGTCCTCTC	ACTGGTAGTCTGCAAAACCAAA
*Acsl1*	TGCCAGAGCTGATTGACATT	GGCATACCAGAAGGTGGTGAG
*Acadm*	AGGGTTTAGTTTTGAGTTGACGG	CCCCGCTTTTGTCATATTCCG
*Cpt1a*	CTCCGCCTGAGCCATGAAG	CACCAGTGATGATGCCATTCT
*Cpt1b*	GCACACCAGGCAGTAGCTTT	CAGGAGTTGATTCCAGACAGGTA
*Cpt1c*	TCTTCACTGAGTTCCGATGGG	ACGCCAGAGATGCCTTTTCC
*Gpat*	ACAGTTGGCACAATAGACGTTT	CCTTCCATTTCAGTGTTGCAGA
*Ucp2*	ATGGTTGGTTTCAAGGCCACA	CGGTATCCAGAGGGAAAGTGAT
*Acox1*	TCCAGACTTCCAACATGAGGA	CTGGGCGTAGGTGCCAATTA
*Me*	GGACTTCTATGACCTGTACGGA	GCTGCGTGTAATACTCGACCA
*Pdk4*	AGGGAGGTCGAGCTGTTCTC	GGAGTGTTCACTAAGCGGTCA
*Homo sapiens*		
*SREBF1*	ACAGTGACTTCCCTGGCCTAT	GCATGGACGGGTACATCTTCAA
*ACC*	ATGTCTGGCTTGCACCTAGTA	CCCCAAAGCGAGTAACAAATTCT
*FASN*	CCGAGACACTCGTGGGCTA	CTTCAGCAGGACATTGATGCC
*FADS2*	GACCACGGCAAGAACTCAAAG	GAGGGTAGGAATCCAGCCATT
*SCD1*	TCTAGCTCCTATACCACCACCA	TCGTCTCCAACTTATCTCCTCC
*SCD2*	CTCTGCGAGTGAATTTGGC	GATCATCGGCTTGGTTGC
*GAPDH*	GCACCGTCAAGGCTGAGAAC	TGGTGAAGACGCCAGTGGA
*KAT2A*	CAGGGCTTCACGGAGATTGT	CTTGGGCACCTTGATGTCCT
*KAT2B*	CTGGAGGCACCATCTCAACGAA	ACAGTGAAGACCGAGCGAAGCA

### CRISPR-Cas9-mediated KAT2A knockout

The KAT2A knockout cell was generated using the CRISPR-Cas9 genome editing system. The sgRNA sequence (5′-ATGGGGCAAACTCTCCAATC-3′) was designed using the Broad Institute CRISPick tool and cloned into the pUC-CBh-gRNA vector, resulting in the pUC-CBh-KAT2A-gRNA construct. HL-7702 cells were transfected with EF1A-GFP-T2A-Puro donor DNA, conferring puromycin resistance, using Lipofectamine 3000 as per the manufacturer's guidelines. Cells underwent 48 h of transfection followed by 72 h of screening with 2 μg/ml puromycin. Single colonies were then isolated, and *KAT2A* knockout was confirmed via western blot analysis.

### Chromatin immunoprecipitation (ChIP) and subsequent sequencing (ChIP-seq) analysis

Chromatin immunoprecipitation (ChIP) was conducted utilizing the SimpleChIP Plus Sonication Chromatin IP Kit (Cell Signaling Technology, 56383S). Cells underwent cross-linking with 1% formaldehyde for 10 min at room temperature, followed by quenching with 0.125 M glycine for 5 min. For tissue samples, approximately 100 mg of liver was minced into 1 mm^3^ pieces and placed in PBS containing a protease inhibitor cocktail before fixation. Cells were rinsed twice with cold PBS, resuspended in PBS with protease inhibitors, and centrifuged at 1000*g* for 5 min at 4 °C. The precipitate was resuspended in Cell Lysis Buffer, and the cells underwent lysis on ice for two 10-min intervals. Nuclei were lysed with Nucleus Lysis Buffer and fragmented using a Branson SFX250 Sonifier at 50% amplitude with a 6-min cycle of 1-s on/off sonic processing. Chromatin was centrifuged at 21,000*g* for 10 min at 4 ℃ and the supernatant was collected. Each 10 μg of chromatin was incubated with 2 μg of the indicated antibody or IgG for 16 h at 4 ℃. The samples underwent a 2-h incubation with Protein G beads at 4 ℃, followed by washing. Chromatin was eluted from the beads and de-crosslinked. After DNA purification, it was relatively quantified by qPCR. Table 2 lists the primers utilized for ChIP-qPCR.

**Table 2:** Primers for ChIP-qPCR

Gene	Forward	Reverse
*Homo sapiens*		
SREBF1c (LXRE)	GAGAACCCGACACGAGGC	TTGCGAGGTTACTCACGGTC
ABCA1 (LXRE)	ATCCCTACCCTTGTGAGCCT	CGAGGTCACTCACTTGGCTT
Mus musculus		
SREBF1c (LXRE)	AGGCTCTTTTCGGGGATGG	TGGGGTTACTGGCGGTCAC
ABCA1 (LXRE)	GGGGAAAGAGGGAGAGAACAG	GAATTACTGGTTTTTGCCGC
SREBF1c (Non-XRE)	TTTGTCATTGGCTGTGGTCTTC	CGGCATGGTCCTGATTGC
ABCA1 (Non-LXRE)	GGCAGTGCCTTTGTAGCCTATG	GGTTCCACACCAGAGTTTCACA

ChIP-seq data were sourced from the GEO database, specifically GCN5 ChIP-seq (GSM1003804, GSE94229) and H3K9ac ChIP-seq (GSM1000141, GSM918712). The adaptor sequence and low-quality fragments were removed from the sequencing data using Skewer (version 0.2.2) and FastQC (version 0.11.5) was applied for quality control analysis. Using Bowtie2 (version 2.5.2), the cleaned reads were aligned to the mm9 mouse genome. MACS2 (v2.1.1) was used for peak calling in tissues to identify protein-DNA interaction sites. ChIP-seq peaks were examined using IGV (v2.17.2). Heatmaps were generated using R version 3.6.1 with the pheatmap package (version 1.0.12).

### Co-immunoprecipitation

For co-IP analysis, cells were lysed on ice for 30 min in 500 µl of lysis buffer containing 50 mM Tris–Cl (pH 7.4), 1 mM EDTA, 0.2% Triton X-100, and a protease inhibitor. Following centrifugation, 50 µl of the supernatant was reserved for western blot analysis. The remaining lysate was incubated overnight at 4 °C with the specific antibody, then with protein A/G agarose beads (Santa Cruz Biotech., sc-2003) for 3 h. The beads/protein complex was washed five times with cold lysis buffer, boiled in 40 µl of SDS loading buffer, and analyzed via western blot.

### Animal

The animal experimental center is accredited by the Association for Assessment and Accreditation of Laboratory Animal Care International. Animals were cared for in accordance with the National Institutes of Health's Guide for the Care and Use of Laboratory Animals. The Animal Ethics Committee of China Pharmaceutical University (2024–06-017) approved all animal experiments and care. Male C57BL/6 J wild-type mice, ob/ob mice, and littermate controls (SPF grade, 6–7 weeks old, 20 to 22 g) were obtained from GemPharmatech Co. Ltd (Nanjing, China). Animals were kept under a 12-h light–dark cycle at temperatures of 22–24 °C, with unrestricted access to water and standard chow (Jiangsu Synergetic Biology Co., Ltd, 1,010,039), unless specified otherwise.

### HFD feeding

To model the progression of metabolic dysfunction-associated steatotic liver disease (MASLD), mouse models were established by administering a high-fat diet (60% fat, 20.6% carbohydrate, 19.4% protein) for 10 weeks. Mice were randomly assigned to different groups and fed either a standard chow diet (Jiangsu Synergetic Biology, 1010039) or a high-fat diet. In addition, they received daily intragastric gavage of either 0.5% carboxymethylcellulose sodium (CMC-Na) or AS-IV suspended in 0.5% CMC-Na.

### Histological analysis of liver

Livers were fixed in 4% paraformaldehyde at 4 °C overnight, embedded in paraffin, and sectioned into 5 μm slices. Liver sections underwent hematoxylin and eosin (H&E) staining to assess morphology. Liver sections underwent oil red O staining and hematoxylin counterstaining to assess lipid accumulation. Liver sections were stained by Sirius Red to assess liver fibrosis. The specified antibodies were used for Immunohistochemistry (IHC) staining. Sections were examined and documented with an Olympus BX53 upright fluorescence microscope (Tokyo, Japan). Lipid droplets were quantified using ImageJ (Version 1.5a).

### Quantification and statistical analysis

GraphPad Prism (Version 10) was used for data analysis. Data are presented as mean ± SEM. The Student's t-test was employed to assess statistical significance between the two groups. When comparison between three or more groups, one-way ANOVA or two-way ANOVA was used. The D'Agostino-Pearson test assessed data conformity to a normal distribution. Statistical significance was defined as *p* < 0.05, with *P < 0.05, **P < 0.01, ***P < 0.001, and 'ns' indicating not significant.

## Results

### AS-IV treatment alleviates hepatic steatosis caused by a high-fat diet both in vitro and in vivo

**Fig. 1:**
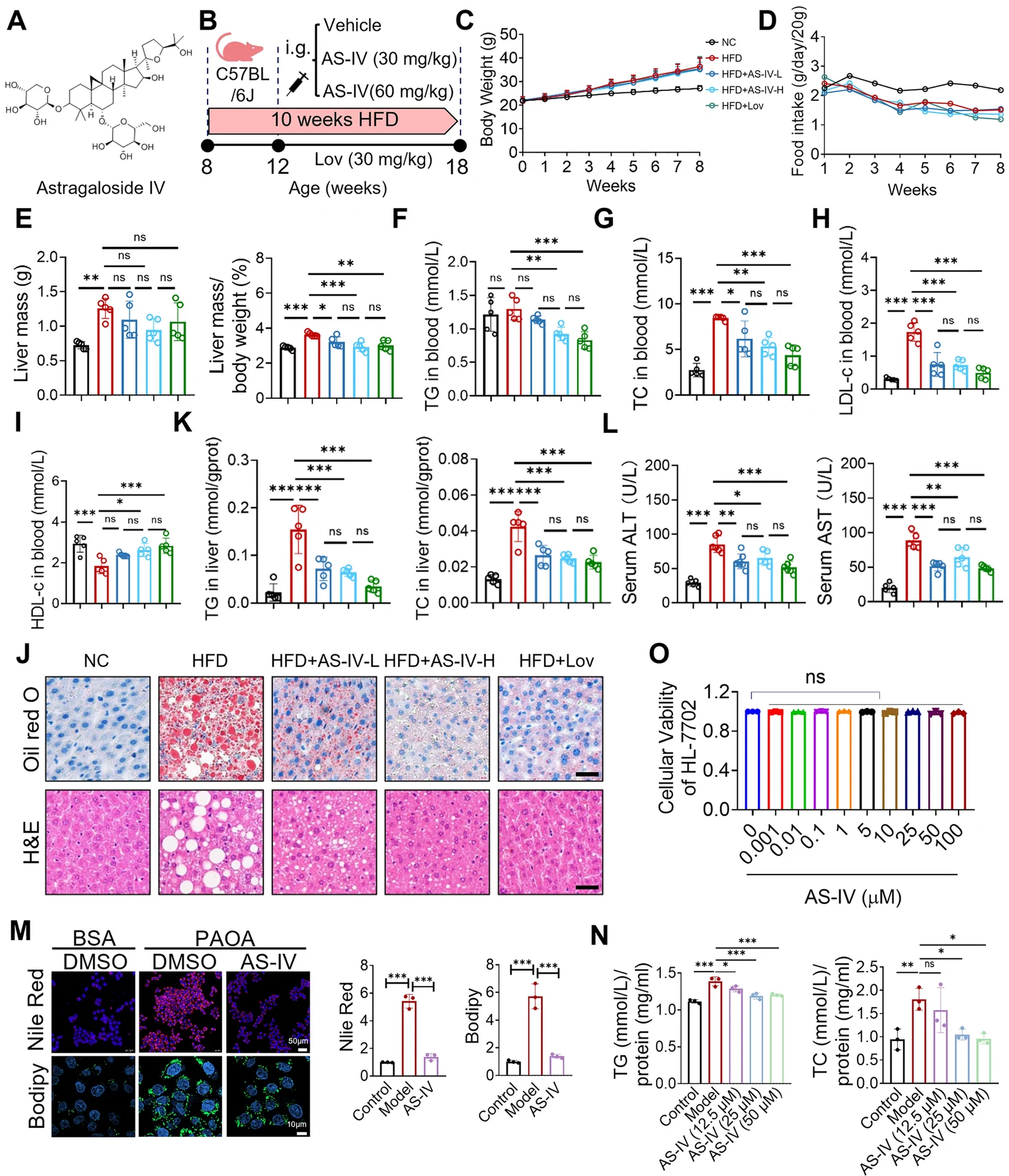
The effects of AS-IV on HFD-induced hepatic steatosis in mice and PA/OA-induced lipid accumulation in hepatocytes. **A** Chemical structure of AS-IV. **B** Schematic of the study design. ND- and HFD-fed mice were treated with vehicle or AS-IV by intragastric gavage for 6 weeks (*n =* 5). **C**, **D** Body weight and food intake. **E** Liver weight (LW) and the ratio of LW and body weight (BW), LW: BW. **F**–**I** The serum TG, TC, LDL-C, and HDL-C levels. **J** Oil red O staining in the liver and histological analysis of the liver. **K** The liver TG and TC levels. **L** The serum ALT and AST levels. **M** HL-7702 cells were incubated PO (PA, 100 μM/OA, 200 μM) with AS-IV (50 μM) for 16 h. The treated HL-7702 cells were fixed and stained with Nile Red and Bodipy. Quantification of the cellular cholesterol or neutral lipids was analyzed by Image-ProPlus (*n =* 3). **N** The cellular TG and TC contents were measured in the treated HL-7702 cells (*n =* 3). **O** The cellular activity was detected by CCK8 (*n =* 3). Error bars are represented as mean ± SEM. Statistical analysis was done with one-way ANOVA (Dunnett’s post-test). *p < 0.05, **p < 0.01 ***p < 0.001

Initially, we examined the impact of AS-IV on lipid accumulation reduction both in vitro and in vivo. To verify AS-IV's regulatory impact on MASLD, mice were administered an HFD and supplemented with or without AS-IV (30 and 60 mg/kg) for 10 weeks (Fig. 1A, B). Throughout the experiment, there was no difference in body weights between AS-IV-treated mice and those fed HFD (Fig. 1C), without food intake change (Fig. 1D). AS‑IV treatment did not significantly affect absolute liver weight (LW) in HFD‑fed mice, but significantly reduce the liver weight‑to‑body weight ratio (LW/BW) (Fig. 1E). AS-IV administration significantly improved serum levels of TG, TC, and the LDL-c/HDL-c ratio in HFD-fed mice compared to HFD-fed controls (Fig. 1F–I). Meanwhile, AS-IV treatment significantly reduced the liver lipid droplets induced by HFD, as demonstrated by H&E and ORO staining of liver sections (Fig. 1J). AS-IV treatment effectively inhibited diet-induced hepatic steatosis, as evidenced by decreased hepatic TG and TC levels (Fig. 1K). Moreover, AS-IV treatment reduced HFD-induced increases in ALT and AST levels, indicating its hepatoprotective effects (Fig. 1L). Moreover, high doses of AS-IV exhibited effects on diet-induced metabolic disorders comparable to lovastatin. The findings suggest that AS-IV effectively addresses metabolic dysfunction, particularly hepatic steatosis, in mice fed a high-fat diet.

Human HL-7702 normal liver cells were exposed to AS-IV (25 and 50 μM) alongside palmitic and oleic acids (PA + OA; PO) for 16 h. Staining with neutral lipid dyes Nile-Red and Bodipy revealed a significant reduction in the size and number of lipid droplets in hepatocytes due to AS-IV treatment (Fig. 1M). The lipid-lowering effect of AS-IV was also validated through the assessment of the cellular TG and TC levels (Fig. 1N, and S1A, B), without significant cytotoxicity (Fig. 1O). These results indicated that AS-IV alleviated lipid accumulation in the free fatty acid (FFA)-induced MASLD cellular model.

### AS-IV treatment alleviates HFD-induced hepatic steatosis through SREBP-1c-mediated DNL

**Fig. 2:**
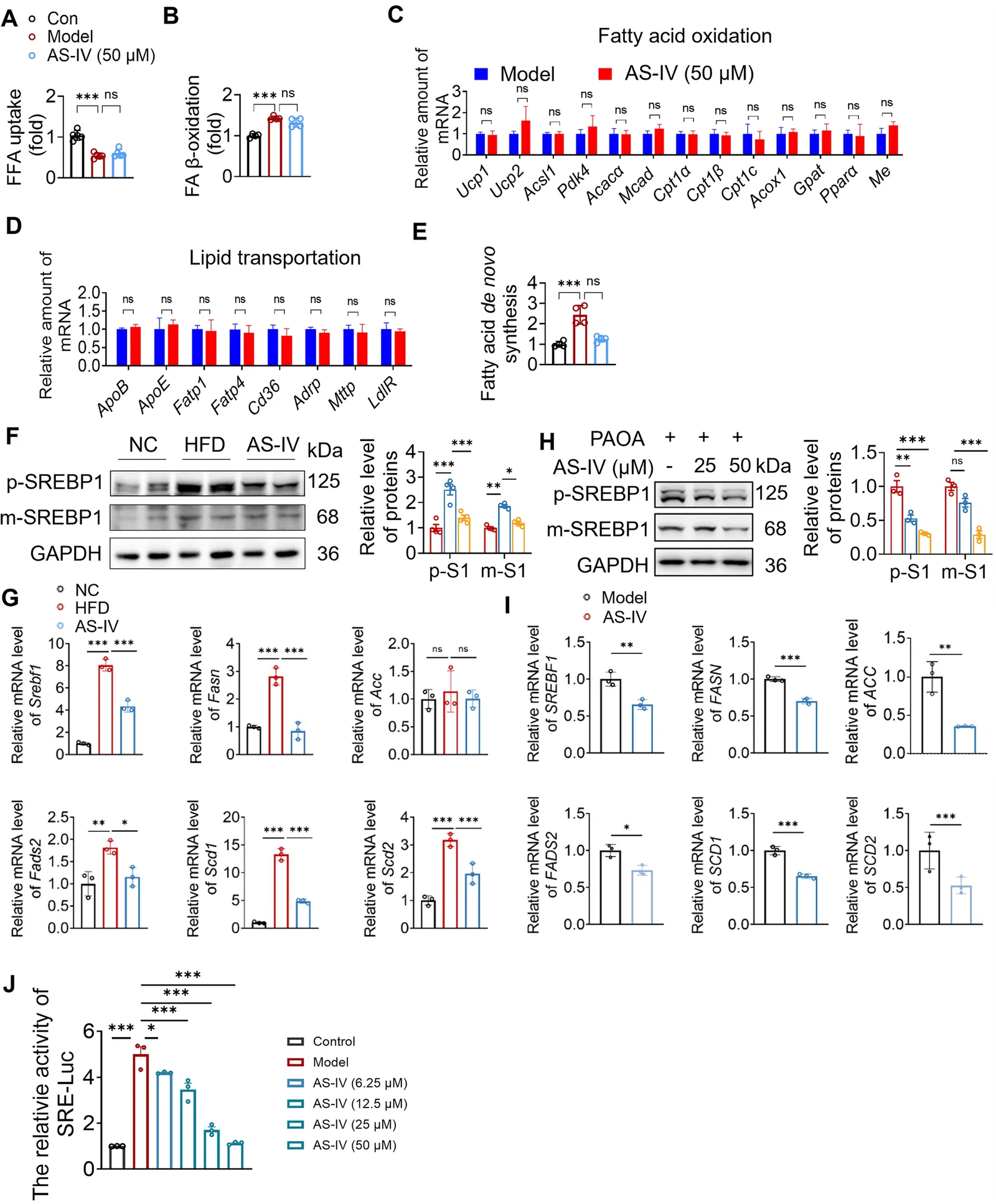
AS-IV treatment ameliorates HFD-induced hepatic steatosis via SREBP-1c-mediated DNL. **A** Free fatty acid (FFA) uptake was measured in the HL-7702 cells (*n =* 4). **B** Oxygen consumption rate (OCR) was measured in the HL-7702 cells (*n =* 4). **C**, **D** The mRNA expression of lipid transportation, and FA oxidation-related genes (*n =* 4). **E** The DNL was measured in the HL-7702 cells (*n =* 4). **F** The protein level of SREBP1 in the liver of HFD mice (*n =* 4). **G** The levels of SREBP1-targeted genes in the liver of HFD mice (*n =* 4). **H** The protein level of SREBP1 in HL-7702 cells. **I** The levels of SREBP1-targeted genes in HL-7702 cells (*n =* 3). **J** The SRE transcriptional activity (*n =* 3). Error bars are represented as mean ± SEM. Statistical analysis was done with one-way ANOVA (Dunnett’s post-test). *p < 0.05, **p < 0.01 ***p < 0.001

To examine AS-IV's regulatory impact on lipid metabolism in hepatocytes, the study focused on the critical processes of FFA uptake and FA β-oxidation. The findings revealed that AS-IV could not change the FA β-oxidation and FFA uptake in hepatocytes (Fig. 2A, B, and S2A, B). AS-IV treatment did not significantly alter the expression levels of genes associated with lipid transportation and fatty acid oxidation (Fig. 2C, D, and S2C, D). AS-IV treatment subsequently reduced de novo lipogenesis (DNL) in the hepatocytes (Fig. 2E). These results suggest that AS-IV may play a lipid-lowering role by decreasing DNL in hepatocytes.

In mammalian cells, SREBPs, comprising the isoforms SREBP1a, SREBP1c, and SREBP2, are key regulators of genes essential for triglyceride and cholesterol synthesis. SREBP2 primarily activates genes for cholesterol synthesis, while SREBP1 enhances transcription of genes associated with TG synthesis. We hypothesize that AS-IV treatment alleviates HFD-induced hepatic steatosis by modulating triglyceride biosynthesis through SREBP-1c-mediated DNL. SREBP1, located in the ER membrane, is activated by converting its precursor (p-SREBP1) into the soluble mature form (m-SREBP1) via a distinct proteolytic pathway. AS-IV treatment in mice significantly reduced the protein levels of SREBP1 (p-SREBP1, and m-SREBP1) and expression of its target genes, such as *Acc*, *Fasn*, *Scd1*, *Scd2*, and *Fads2* (Fig. 2F, and G). AS-IV treatment in hepatocytes significantly reduced the protein levels of p-SREBP1 and m-SREBP1 (Fig. 2H, and S2E), and SREBP1c target genes, including *SREBF1*, *ACC*, *FASN*, *SCD1*, *SCD2*, and *FADS2* (Fig. 2I). Moreover, AS-IV also inhibited SREBP transcriptional activity in hepatocytes (Fig. 2J). Altogether, these findings indicate that AS-IV treatment alleviates HFD-induced hepatic steatosis through SREBP-1c-mediated DNL. Given that LXRα is a master transcriptional activator of SREBP-1c, we next examined whether AS-IV modulates the GCN5‑LXRα axis to exert its anti‑lipogenic effects.

### The effect of AS-IV in hepatocytes was dependent on SREBP1c.

**Fig. 3:**
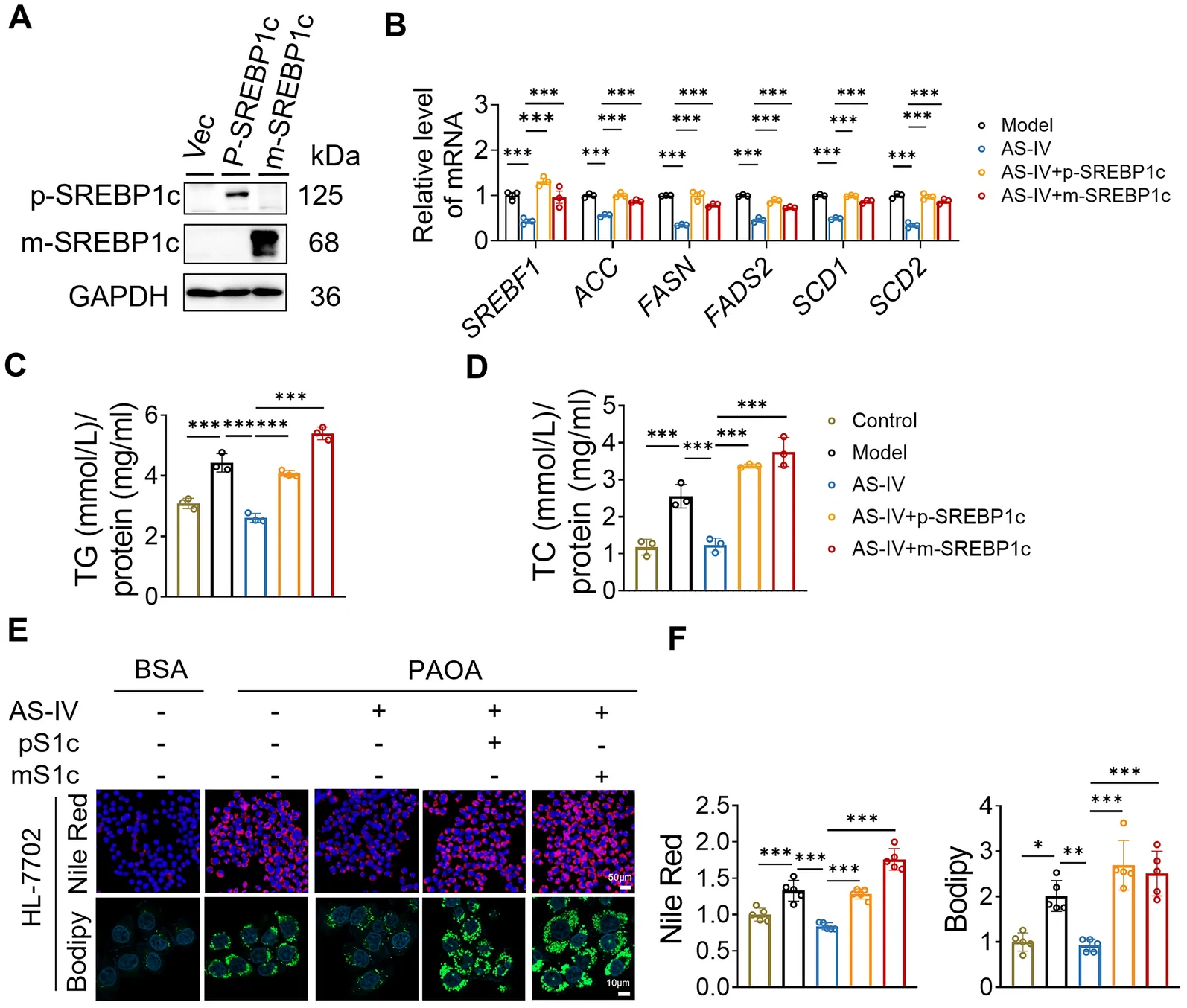
Overexpression of SREBP-1c reversed the effect of AS-IV in hepatocytes. HL-7702 cells were transfected with the indicated plasmids for 24 h. Cells were incubated PO (PA, 100 μM/OA, 200 μM) with AS-IV (50 μM) for 16 h. **A** The protein expression of SREBP1 was detected by western blot (*n =* 3). **B** The levels of SREBP1-targeted genes in HL-7702 cells (*n =* 3). **C**, **D** The cellular TG and TC contents were measured in the treated HL-7702 cells (*n =* 3). **E**, **F** The treated HL-7702 cells were fixed and stained with Nile Red and Bodipy (*n =* 5). Quantification of cellular cholesterol and neutral lipids was analyzed using Image-ProPlus. Error bars are represented as mean ± SEM. Statistical analysis was done with one-way ANOVA (Dunnett’s post-test). *p < 0.05, **p < 0.01 ***p < 0.001

To verify that AS-IV's protective effect operates via the SREBP1 pathway, we overexpressed p-SREBP1c or m-SREBP1c in hepatocytes and conducted in vitro assays for PO-induced lipid accumulation (Fig. 3A). Our findings indicate that SREBP1c rescue significantly upregulated the DNL-related genes (Fig. 3B), and reversed the suppressive effect of AS-IV on cellular TG and TC content (Fig. 3C, D). Moreover, the overexpression of p-SREBP1c and m-SREBP1c counteracted the suppressive impact of AS-IV on the SREBP pathway in HL-7702 cells in vitro (Fig. 3C, D). Staining hepatocyte lipid droplets with neutral lipid dyes Nile-Red and Bodipy revealed that AS-IV reduced lipid droplet accumulation, including size and number, an effect reversed by overexpressing p-SREBP1c and m-SREBP1c (Fig. 3E, F). Moreover, overexpression of p-SREBP1c and m-SREBP1c rescued the expression of DNL-related genes significantly suppressed by AS-IV. We then confirmed the efficacy of the SREBP-1c knockdown model (Fig. S3A). Subsequent experiments demonstrated that upon SREBP-1c knockdown, the upregulation of key downstream lipogenic genes, such as *ACC*, was completely abolished (Fig. S3B). Accordingly, the inhibitory effects of AS-IV on cellular TG and TC content were no longer observed in the absence of SREBP-1c (Fig. S3C, D). This phenotypic reversal was further confirmed at the morphological level: Nile Red and Bodipy staining revealed that lipid droplet accumulation remained consistently low following SREBP-1c knockdown, regardless of AS-IV treatment (Fig. S3E, F). These data collectively demonstrated that SREBP1c is critical for the anti-MASLD progression effect of AS-IV.

### AS-IV inhibited SREBP1c-mediated DNL dependent on GCN5

**Fig. 4:**
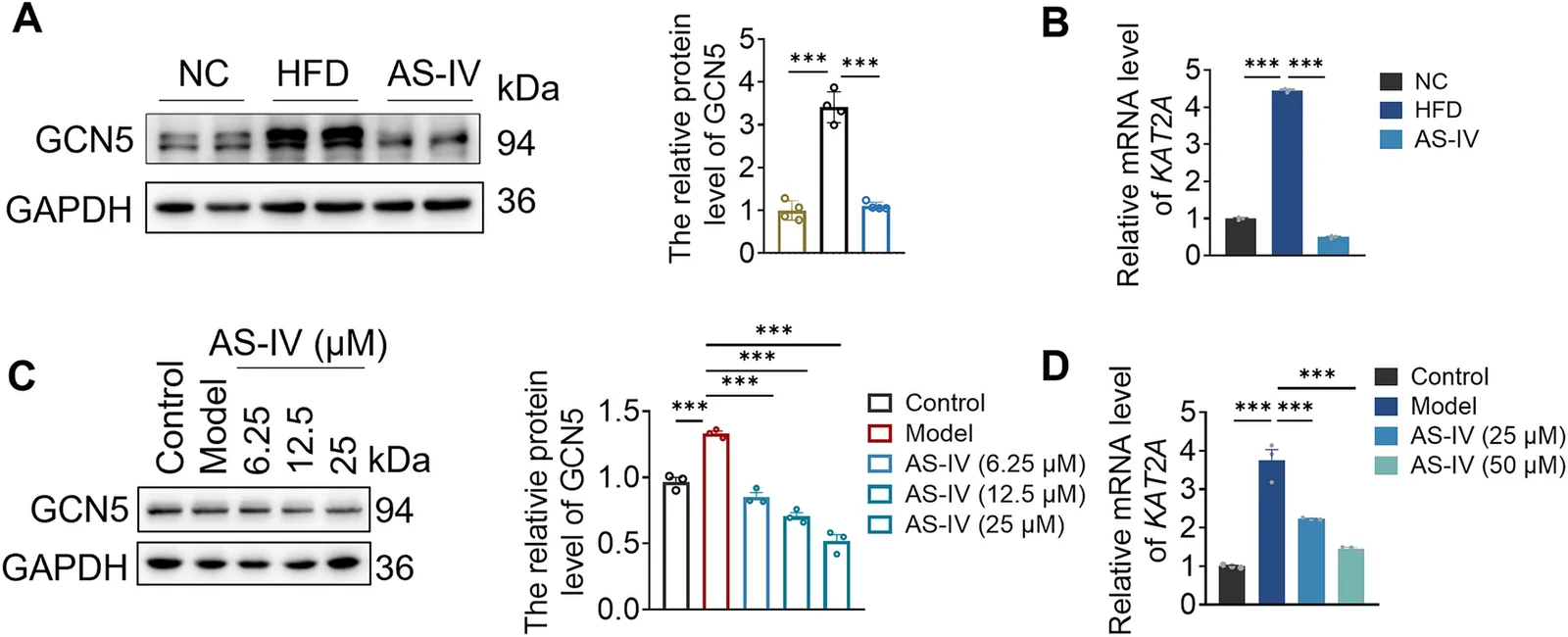
Hepatocyte GCN5 is overexpressed in MASLD models. **A**, **B** The protein and mRNA expression of GCN5 in the livers of HFD-induced MALFD (*n =* 4). **C**, **D** HL-7702 cells were incubated PO (PA, 100 μM/OA, 200 μM) with AS-IV (50 μM) for 16 h. The protein and mRNA expression of GCN5 in the above cells (*n =* 3). Error bars are represented as mean ± SEM. Statistical analysis was done with one-way ANOVA (Dunnett’s post-test). *p < 0.05, **p < 0.01 ***p < 0.001

We initially assessed GCN5 expression in HFD-induced MASLD to explore its role in hepatic lipid metabolism and modulation by AS-IV. Western blot and q-PCR analyses revealed that HFD-fed mice exhibited a significant upregulation of GCN5 protein (3.2-fold increase) and mRNA levels (4.4-fold increase) in liver tissues compared to chow diet-fed controls (Fig. 4A, B). Strikingly, AS-IV treatment normalized these elevations, reducing GCN5 protein and mRNA expression by 58% and 65%, respectively (Fig. 4A, B).

To validate these findings in vitro, we exposed human hepatocyte lines to PO to mimic lipid overload. Consistent with in vivo results, PO stimulation significantly increased GCN5 protein and mRNA levels compared to untreated cells (Fig. 4C, D). AS-IV administration dose-dependently attenuated these effects, with 25 μM AS-IV reducing GCN5 protein by 52% in HL-7702 cells, and mRNA levels by 60%, respectively (Fig. 4C, D). These results collectively demonstrate that AS-IV robustly inhibits GCN5 expression under lipid-overloaded conditions, implicating GCN5 as a critical mediator of AS-IV’s anti-steatotic effects.

**Fig. 5:**
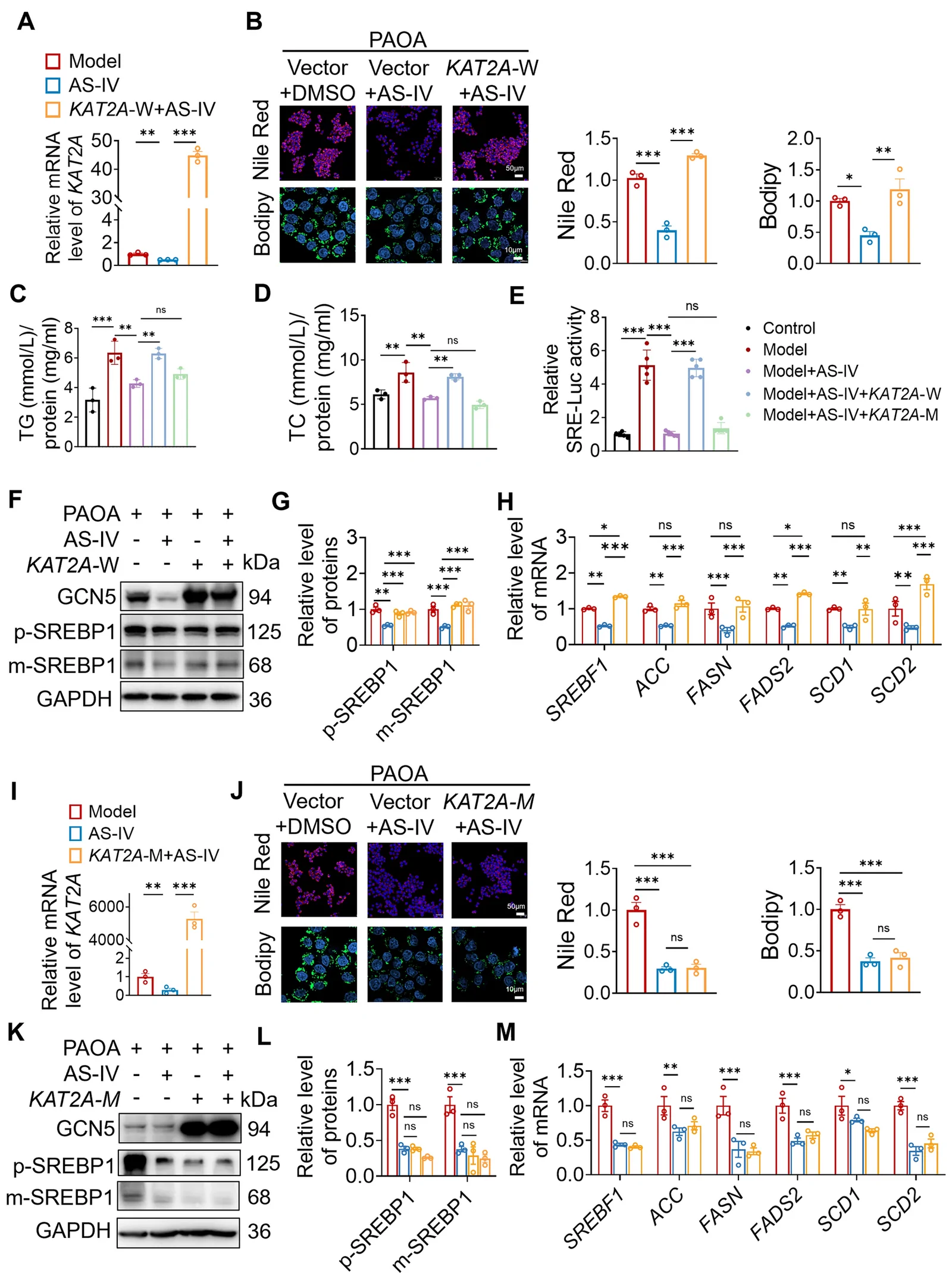
The effect of AS-IV on the SREBP-1 pathway dependent of GCN5. HL-7702 cells were transfected with the GCN5 WT plasmids for 24 h. Cells were incubated PO (PA, 100 μM/OA, 200 μM) with AS-IV (50 μM) for 16 h. **A**, **B** The treated HL-7702 cells were fixed and stained with Nile Red and Bodipy. Quantification of cellular cholesterol and neutral lipids was analyzed using Image-ProPlus (*n =* 3). **C**, **D** The cellular TG and TC contents were measured in the treated HL-7702 (*n =* 3). **E** The SRE transcriptional activity (*n =* 5). **F**–**G** The protein expression of indicated proteins was detected by western blot (*n =* 3). **H** The levels of SREBP1-targeted genes in HL-7702 cells. HL-7702 cells were transfected with the GCN5 MUT plasmids for 24 h. Cells were incubated PO (PA, 100 μM/OA, 200 μM) with AS-IV (50 μM) for 16 h (*n =* 3). **I**, **J** The treated HL-7702 cells were fixed and stained with Nile Red and Bodipy. Quantification of cellular cholesterol and neutral lipids was analyzed using Image-ProPlus (*n =* 3). **K**, **L** The protein expression of indicated proteins was detected by western blot (*n =* 3). **M** The levels of SREBP1-targeted genes in HL-7702 cells (*n =* 3). Error bars are represented as mean ± SEM. Statistical analysis was done with one-way ANOVA (Dunnett’s post-test). *p < 0.05, **p < 0.01 ***p < 0.001

To confirm the necessity of GCN5 in mediating AS-IV’s anti-lipogenic effects, we generated a GCN5-overexpressing plasmid (*KAT2A-WT*) and transfected it into hepatocytes. In GCN5-overexpressing cells, AS-IV failed to reduce lipid droplet accumulation, as evidenced by unaltered Nile Red and Bodipy fluorescence intensities (Fig. 5A, B). Similarly, AS-IV no longer lowered cellular TG or TC levels (Fig. 5C, D). Moreover, AS-IV lost its ability to suppress SREBP1c transcriptional activity (Fig. 5E), as shown by unaltered precursor and mature SREBP1c protein levels (Fig. 5F, G) and persistent expression of downstream lipogenic genes (Fig. 5H).

To dissect the role of GCN5’s acetyltransferase activity, we introduced an acetyltransferase-defective mutant (GCN5-E575Q). In GCN5-E575Q-overexpressing cells, AS-IV retained its efficacy, reducing lipid droplet accumulation (Fig. 5I, J), and TG and TC levels (Fig. 5C, D). Moreover, AS-IV retains its ability to suppress SREBP-1c transcriptional activity (Fig. 5E), as shown by unaltered precursor and mature SREBP-1c protein levels (Fig. 5K, L) and persistent expression of downstream lipogenic genes (Fig. 5M), indicating that AS-IV’s effects depend on GCN5’s acetyltransferase activity.

**Fig. 6:**
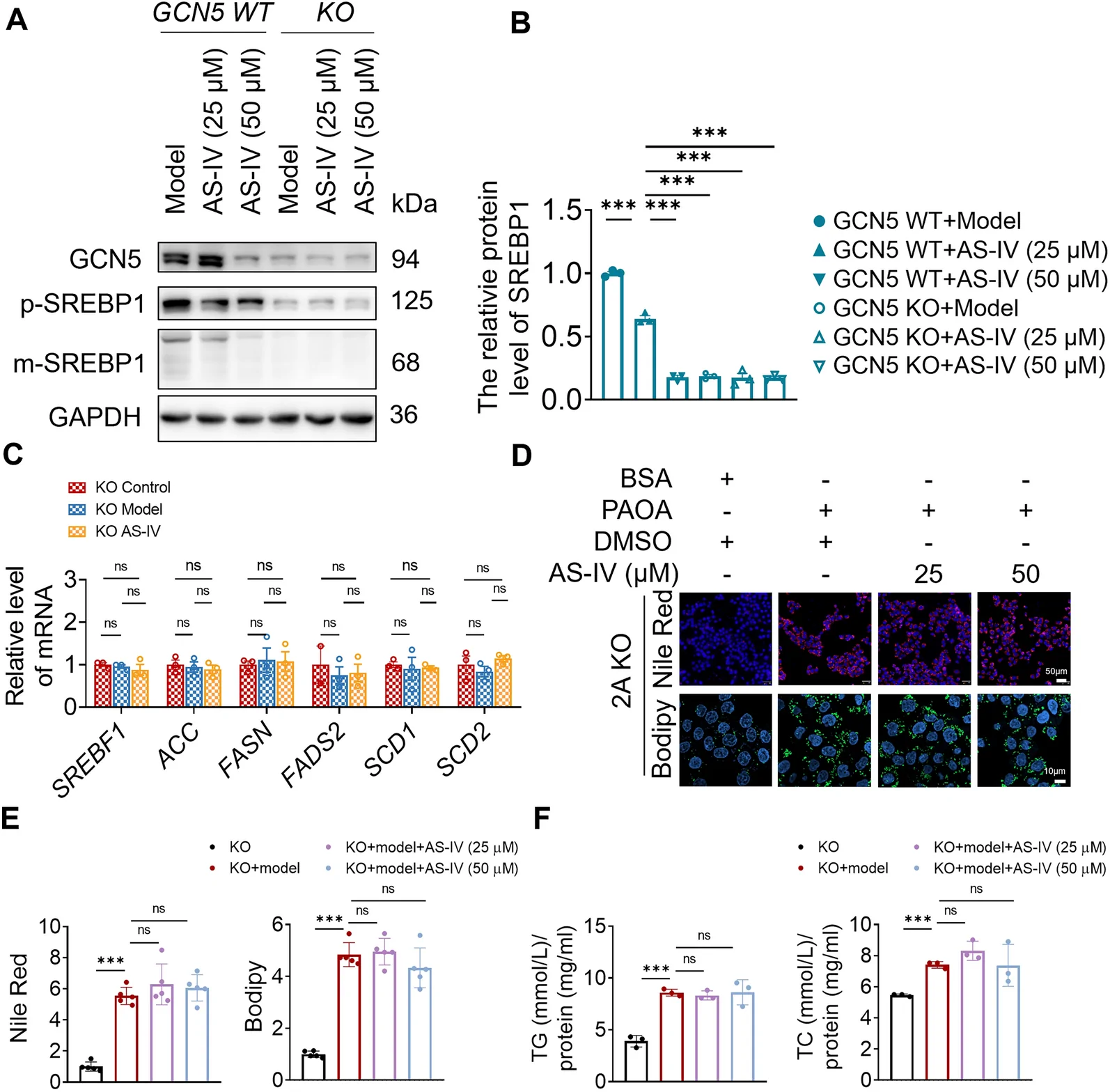
The effect of AS-IV on the SREBP-1 pathway in GCN5 KO HL-7702 cells. HL-7702 and GCN5 KO HL-7702 cells were incubated PO (PA, 100 μM/OA, 200 μM) with AS-IV (50 μM) for 16 h. **A**, **B** The expression of indicated proteins was detected by western blot (*n =* 3). **C** The mRNA levels of SREBP1-targeted genes (*n =* 3). **D**, **E** The treated HL-7702 cells were fixed and stained with Nile Red and Bodipy. Quantification of the cellular cholesterol or neutral lipids was analyzed by Image-ProPlus (*n =* 5). **F** The cellular TG and TC contents were measured in the treated GCN5 KO HL-7702 cells (*n =* 3). Error bars are represented as mean ± SEM. Statistical analysis was done with one-way ANOVA (Dunnett’s post-test). *p < 0.05, **p < 0.01 ***p < 0.001

To further define the dependency of AS-IV on GCN5 in regulating SREBP1c-mediated DNL, we examined the SREBP1c pathway in GCN5 KO HL-7702 cells. In wild-type (WT) hepatocytes, AS-IV significantly reduced PO-induced SREBP1c precursor and mature protein levels (Fig. 6A, B), Strikingly, AS-IV treatment in KAT2A-KO cells failed to further suppress SREBP1c or its target genes (Fig. 6A–C). In line with these molecular alterations, AS-IV was ineffective in decreasing PO-induced lipid droplet accumulation in KAT2A-KO cells, as measured by Nile Red and Bodipy fluorescence (Fig. 6D, E). Similarly, cellular TG and TC levels remained unaltered after AS-IV treatment (Fig. 6F). These data unequivocally demonstrate that AS-IV inhibits SREBP1c-mediated hepatic DNL in a GCN5 acetyltransferase activity-dependent manner.

### GCN5 epigenetics promotes SREBP1c transcriptional expression

**Fig. 7:**
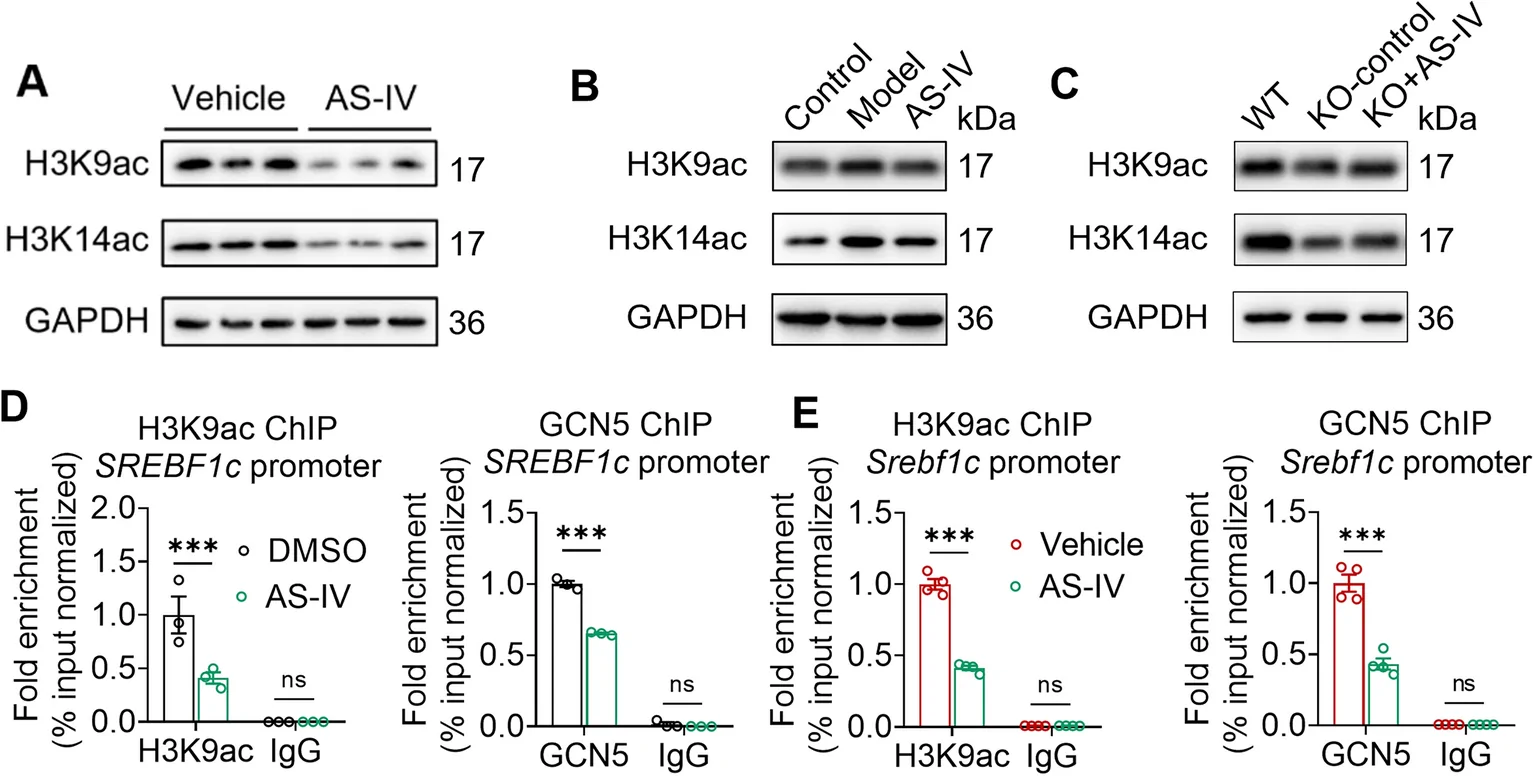
AS-V epigenetically promotes SREBP1c transcriptional expression. **A** The protein levels of H3K9ac and H3K14ac in the fatty liver of HFD-induced mice (*n =* 3). **B**, **C** HL-7702 (GCN5 WT or KO) cells were treated with AS-IV (50 μM) for 24 h. The protein levels of H3K9ac and H3K14ac were detected. **D** H3K9ac and GCN5 recruitment to the promoter of the SREBF1 gene were detected by ChIP-qPCR (*n =* 3). **E** HFD diet-induced NAFLD models treated with AS-IV. The liver H3K9ac and GCN5 recruitment to the promoter of the SREBF1 gene were detected by ChIP-qPCR (*n =* 4). Error bars are represented as mean ± SEM. Statistical analysis was done with one-way ANOVA (Dunnett’s post-test). *p < 0.05, **p < 0.01, ***p < 0.001

Having established that AS-IV inhibits GCN5 and that GCN5 controls SREBP-1c transcription through histone acetylation, we next asked whether this regulation converges with the LXRα pathway. We propose that GCN5 epigenetically regulates the SREBP1 pathway. GCN5, a histone acetyltransferase, modulates the acetylation of histone 3 at lysines 9 and 14. Our study revealed that in MASLD, elevated levels of H3K9ac and H3K14ac were observed, which showed a positive correlation with increased GCN5 expression (Fig. 7A). In vitro, PAOA modeling also increased H3K9ac and H3K14ac levels (Fig. 7B). Subsequently, when AS-IV was applied to hepatocytes or mice to inhibit GCN5 activity, the levels of H3K9ac and H3K14ac proteins in hepatocytes and liver were also significantly reduced (Fig. 7A, B). However, in KAT2A KO hepatocytes, AS-IV lost its ability to down-regulate the expression of GCN5 (Fig. 7C). The above experimental results proved that GCN5 participated in the transcriptional regulation of SREBP1c, so GCN5 should be able to be recruited into the promoter region of SREBF1c. We employed ChIP to verify the hypothesis by examining GCN5 recruitment at the SREBF1c gene promoter. As shown in Fig. 7D, E, GCN5, and H3K9ac could be recruited to the promoter region of the SREBF1c gene in HL-7702 cells and the liver tissue of MASLD. We employed AS-IV to inhibit GCN5 activity to examine HAT's role in regulating SREBF1c expression. The results showed that AS-IV could significantly inhibit the interaction of GCN5 and H3K9ac with the SREBF1c promoter region (Fig. 7D, E). In summary, GCN5 enhances SREBF1c gene expression by modifying histone H3 acetylation in the SREBF1c promoter via its acetyltransferase function. AS-IV epigenetically suppresses SREBF1c expression in a GCN5-dependent manner.

### AS-IV inhibits the transcriptional expression and activity of LXRα/RXRα-mediated SREBP1c dependent on GCN5

**Fig. 8:**
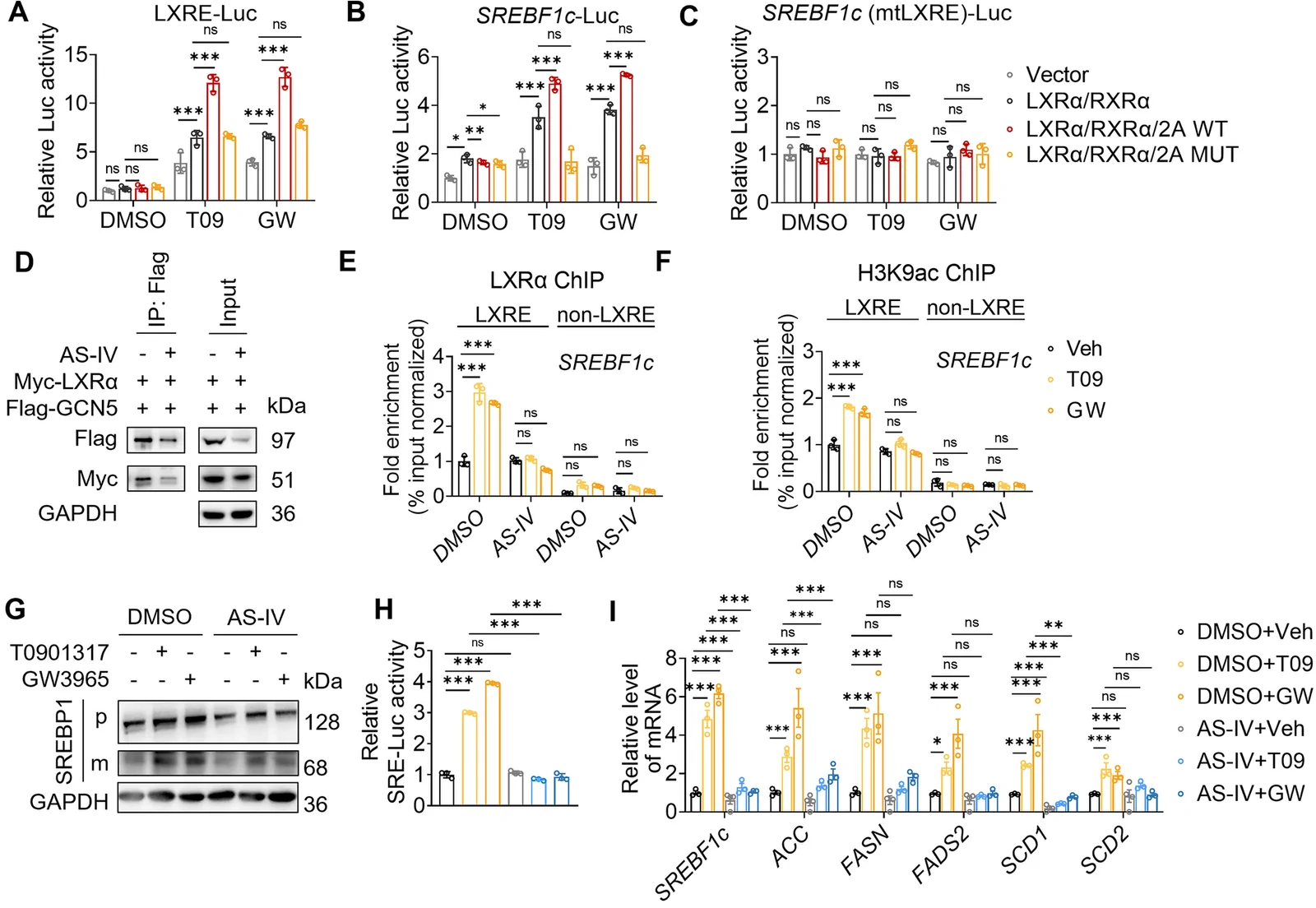
AS-IV selectively stimulates SREBP1c activity in a ligand-dependent manner via LXRE. **A**–**C** HL-7702 cells were transfected with LXRα/RXRα/GCN5, and a luciferase reporter plasmid under the control of synthetic LXRE (A), *SREBF1c* WT promoter (**B**), *SREBF1c Mutant* promoter (**C**), incubated with T0901317 (T09, 1 μM) and GW3965 (GW, 1 μM) (*n* = 3). **D** HL-7702 cells were transfected with Myc-tag-LXRα and Flag-tag*-*GCN5 plasmids for 24 h, and treated with AS-IV for 24 h. The interaction of GCN5 and LXRα was detected by IP. **E**, **F** HL-7702 cells were treated AS-IV with T0901317 and GW3965 for 24 h. ChIP assays were performed with anti-LXRα (**E**) or anti-H3K9ac (**F**) antibodies. Recruitment of LXRα and H3K9ac to the LXREs of the *SREBF1c* gene was determined by qRT-PCR (*n =* 3). **G**–**I** HL-7702 cells were treated AS-IV with T0901317 and GW3965 for 16 h (*n =* 3). **G** The protein levels of SREBP1 were detected by western blot. **H** The SRE-Luc activity was detected by luciferase assay. **I** The indicated gene expression was detected by qRT-PCR. Error bars are represented as mean ± SEM. Statistical analysis was done with one-way ANOVA (Dunnett’s post-test). *p < 0.05, **p < 0.01, ***p < 0.001

Earlier studies have indicated that the liver X receptor (LXR) activates the transcription of SREBP1c. We conducted cotransfection and luciferase reporter assays to investigate the collaborative role of GCN5 with LXRα/RXRα in enhancing SREBP1c transcription and expression. *KAT2A* enhanced the LXRα’s transcriptional activity on a synthetic LXRE promoter in a ligand-dependent manner using T0901317 (Fig. 8A). We also investigated their coactivator roles in two physiologically significant natural promoters with LXRE. The findings indicated that *KAT2A* WT enhanced the transcriptional activity of the *SREBF1c* promoter (Fig. 8B). Significantly, *KAT2A* WT failed to activate transcription of *SREBF1c* promoters with LXRE mutations (Fig. 8C), demonstrating that their coactivator role relies specifically on LXR binding to the LXRE. The target gene-specific activity of GCN5 was also investigated using GW3965, a distinct LXR ligand structurally different from T090137.GCN5 selectively activated the *SREBF1c* promoter through LXR mediation when influenced by GW3965 (Fig. 8B, C). Cotransfection of Flag‑tagged GCN5 and Myc‑tagged LXRα in HL‑7702 cells, followed by co‑immunoprecipitation (Co‑IP), confirmed a strong interaction between GCN5 and LXRα; reciprocal Co‑IP further validated this association (Fig. 8D). To determine whether the AS‑IV‑induced reduction in this interaction was due to decreased GCN5 protein abundance rather than direct interference, we performed Co‑IP under normalized GCN5 input conditions—i.e., equal amounts of GCN5 protein were used for immunoprecipitation across all samples. Under this normalized setting, AS‑IV still markedly reduced the co‑immunoprecipitated LXRα (Fig. 8D), indicating that AS‑IV directly disrupts the physical GCN5–LXRα interaction, independent of its effect on GCN5 protein levels.

We conducted ChIP analyses on the *SREBF1c* promoter regions to study GCN5 recruitment in endogenous chromatin. Treatment with T0901317 or GW3965 led to the recruitment of GCN5 and H3K9ac to the LXRE of the endogenous *SREBF1c* promoter, a process inhibited by AS-IV (Fig. 8E, F).

We conducted ChIP assays with antibodies targeting LXRα and H3K9ac to explore GCN5's involvement in activating the *SREBF1c* promoter. AS-IV eliminated the recruitment of LXRα and H3K9ac to the LXRE of the *SREBF1c* promoter induced by T0901317 (Fig. 8E, F). AS-IV also notably reduced the GW3965-induced recruitment of LXRα and H3K9ac to the LXRE of the *SREBF1c* promoter (Fig. 8E, F). GW3965 and T0901317 markedly elevated *SREBF1c* mRNA expression levels (Fig. 8I). However, AS-IV significantly suppressed the T0901317 and GW3965-dependent increase of *SREBF1c* transcription (Fig. 8I). Furthermore, in KAT2A knockout hepatocytes, the capacities of GW3965 and T0901317 to elevate SREBP1 protein levels and transcriptional activity were markedly reduced (Fig. 8G–H). Concurrently, the upregulation of downstream target genes, including *ACC, FASN, SCD1, SCD2*, and *FADS2*, induced by these LXR agonists was also largely abrogated (Fig. 8I). These findings collectively indicate that AS‑IV suppresses LXRα/RXRα‑mediated SREBP1c transcription and activity in a GCN5‑dependent manner.

## Discussion

The present study demonstrates that AS-IV, a bioactive saponin derived from *Astragalus membranaceus*, ameliorates hepatic steatosis in MASLD models by specifically suppressing SREBP1c-mediated DNL. Importantly, we identify a novel epigenetic mechanism wherein AS-IV exerts its effects by inhibiting the histone acetyltransferase GCN5 (KAT2A), thereby reducing histone acetylation at the SREBF1c promoter and disrupting the transcriptional cooperation between GCN5 and liver X receptor alpha (LXRα). Our findings not only confirm the central role of SREBP-1c in hepatic lipid accumulation but also delineate a unique, pathway-selective mode of action for AS-IV that distinguishes it from other natural compounds or therapeutic strategies.

SREBP1c is a well-established driver of DNL, activating lipogenic enzymes such as ACC, FAS, and SCD1. Its overexpression in MASLD has been consistently reported in both preclinical models and human patients. For instance, a study revealed that glycerol kinase (GK) promotes NAFLD progression by directly binding to the SREBP-1c promoter to enhance its transcription, leading to increased TG synthesis [19]. Similarly, resveratrol alleviates hepatic steatosis by downregulating SREBP-1c expression via AMPK/SIRT1 activation, thereby reducing FAS and ACC activity [20]. Our data corroborate these observations, as AS-IV significantly suppressed both precursor and mature forms of SREBP-1c in hepatocytes. However, unlike GK or resveratrol, which act through transcriptional or post-translational modulation, AS-IV uniquely targets the epigenetic regulation of SREBP-1c via GCN5 inhibition, expanding the mechanistic repertoire for SREBP-1c modulation.

Histone acetylation, governed by acetyltransferases like GCN5, plays a pivotal role in transcriptional activation [21, 22]. GCN5-mediated acetylation of histones H3K9 and H3K14 facilitates chromatin relaxation, enabling transcription factors such as LXRα to access the SREBP-1c promoter. Our study provides the first evidence that AS-IV inhibits GCN5 expression, thereby reducing histone acetylation at the SREBP-1c locus and suppressing its transcriptional activity. This mechanism diverges from classic AMPK-dependent pathways, as seen in resveratrol, berberine, or mangiferin, which rely on AMPK phosphorylation to indirectly inhibit SREBP-1c [23–25]. Notably, the rescue experiments—where GCN5 overexpression reversed AS-IV’s effects—confirm the centrality of this epigenetic axis. These findings parallel recent work on GK, where enzyme-independent transcriptional activation of SREBP-1c was observed [19], suggesting that multiple upstream regulators converge on SREBP-1c through distinct mechanisms.

Current MASLD therapies, such as vitamin E or thiazolidinediones, exhibit limited efficacy and safety concerns [26, 27]. Natural compounds like resveratrol and AS-IV offer alternatives by targeting lipid metabolism at multiple nodes. While resveratrol enhances AMPK/SIRT1 signaling to inhibit SREBP-1c [20], AS-IV operates through GCN5-LXRα-SREBP-1c axis, bypassing energy-sensing pathways. This distinction may explain AS-IV’s superior efficacy in our high-fat diet models, particularly in contexts of insulin resistance where AMPK activity is often blunted. Furthermore, unlike GK inhibitors, which primarily reduce TG synthesis by blocking glycerol metabolism [19], AS-IV simultaneously suppresses both DNL and cholesterol synthesis pathways, as evidenced by reduced FAS and HMGCR expression (unpublished data). This dual action positions AS-IV as a multifaceted agent for MASLD intervention.

The translational potential of AS-IV lies in its ability to address the epigenetic dysregulation prevalent in MASLD. GCN5 upregulation has been implicated in obesity-related metabolic disorders, and its inhibition could synergize with lifestyle interventions to mitigate hepatic lipid accumulation [28, 29]. However, challenges remain, including optimizing bioavailability and verifying efficacy in human trials. Future studies should explore combinatorial therapies, such as AS-IV with AMPK activators, to amplify anti-steatotic effects. Additionally, investigating GCN5’s role in other metabolic tissues (e.g., adipose or muscle) could unveil systemic benefits beyond the liver.

We fully agree with the importance of validating key findings in human MASLD liver samples. In a parallel study from our group, we have systematically examined GCN5, LXRα, SREBP1c expression and histone acetylation levels in 117 human MASLD liver tissues and 11 healthy controls [12]. That study demonstrated that GCN5 expression and H3K9ac/H3K14ac levels are significantly elevated in MASLD patients and correlate positively with disease severity and lipid metabolism parameters, establishing the clinical relevance of the GCN5–LXRα–SREBP1c axis in human MASLD.

Nevertheless, we acknowledge that the present study did not directly re‑measure these markers in human samples, and this represents a limitation of the current work.

We acknowledge that the present study did not include dedicated PK or chronic toxicity studies for AS‑IV. However, the selected doses (30/60 mg/kg) and concentrations (25/50 μM) are supported by literature demonstrating efficacy, safety, and hepatic enrichment of AS‑IV [18, 30]. We also observed dose‑dependent trends in both cellular and animal experiments. Nevertheless, future studies should include comprehensive PK and long‑term toxicity evaluation to support clinical translation.

However, several questions arise from our findings that warrant further investigation. First, the selectivity for DNL over FA oxidation is notable. While we show AS-IV does not affect the expression of key β-oxidation genes, the precise reason for this pathway specificity remains unclear. It is possible that the GCN5-LXRα axis has a more dominant role in regulating lipogenic versus catabolic gene programs, or that other compensatory transcription factors (e.g., PPARα) maintain oxidation pathways. Second, while we establish that GCN5's acetyltransferase activity is crucial, the exact substrate profile relevant to SREBP-1c transactivation—whether it involves histones exclusively or also non-histone proteins like LXRα itself-needs elucidation. Third, our data show AS-IV lowers both TG and cholesterol synthesis markers. The reduction in SREBP-1c target genes explains the TG effect, but the observed improvement in serum LDL-c/HDL-c ratio suggests a possible impact on SREBP-2 or other cholesterol regulatory pathways, which was not the focus here and merits separate study.

The translational potential of AS-IV is promising, as it addresses the epigenetic driver (GCN5 upregulation) of metabolic disease. However, limitations exist. The pharmacokinetics and optimal dosing of AS-IV in humans require thorough investigation. Furthermore, the long-term consequences of GCN5 inhibition in extrahepatic tissues need evaluation, given its broad role in gene regulation. Future studies should validate this axis in human primary hepatocytes from patients with hepatic steatosis and explore combinatorial therapies, such as coupling AS-IV with agents that enhance fatty acid oxidation (e.g., PPARα agonists), to potentially achieve synergistic clearance of hepatic fat.

Our study demonstrates that AS-IV inhibits hepatic steatosis by suppressing GCN5 expression, which in turn reduces histone acetylation at the SREBF1c promoter and disrupts LXRα-mediated transcriptional activation of SREBP1c. Importantly, we have shown that GCN5 is not a direct binding target of AS-IV; rather, AS-IV downregulates GCN5 at the transcriptional level. This distinction is critical for understanding the mechanistic hierarchy: GCN5 acts as a downstream effector that is necessary for the anti-lipogenic effects of AS-IV. Such indirect modulation is common among natural products, which often exert their effects through network regulation rather than single-target inhibition. The reliance on GCN5 as a key mediator, despite the absence of direct binding, underscores the importance of this epigenetic regulator in MASLD pathogenesis and highlights the potential of targeting its expression or activity for therapeutic benefit.

In summary, AS-IV represents a novel epigenetic modulator of SREBP-1c, offering a promising strategy for MASLD treatment. By inhibiting GCN5 and reducing histone acetylation at the SREBP-1c promoter, AS-IV disrupts lipogenic programming while avoiding the pitfalls of direct SREBP-1c targeting. These findings not only advance our understanding of MASLD pathogenesis but also highlight the therapeutic potential of natural compounds in metabolic diseases.

## Limitations

Our study has limitations. First, the reliance on rodent models necessitates validation in human hepatocytes or clinical cohorts. Second, while we identified GCN5 as a key mediator, its interaction with other histone modifiers (e.g., HDACs) warrants further exploration. Lastly, the precise molecular link between GCN5 inhibition and LXRα transcriptional suppression remains to be elucidated.

## Conclusion

This study demonstrates that AS-IV ameliorates hepatic steatosis in metabolic dysfunction-associated fatty liver disease (MASLD) by epigenetically suppressing SREBP1c-mediated de novo lipogenesis. Mechanistically, AS-IV downregulates the histone acetyltransferase GCN5 (KAT2A), thereby reducing H3K9ac and H3K14ac enrichment at the SREBF1c promoter and disrupting GCN5–LXRα interaction, which ultimately inhibits LXRα/RXRα-driven SREBP1c transcription. Notably, the anti-lipogenic effects of AS-IV are dependent on GCN5, as genetic ablation or acetyltransferase-deficient mutation of GCN5 abolishes its efficacy. Collectively, our findings uncover a previously unrecognized epigenetic axis-GCN5-LXRα–SREBP1c—that governs hepatic lipogenesis, and position AS-IV as a promising natural epigenetic modulator for MASLD therapy, acting through transcriptional downregulation of GCN5 rather than direct enzymatic inhibition.

## Supplementary Information

Additional file 1.

## Data Availability

No datasets were generated or analysed during the current study.

## Abbreviations

ABCA1: ATP-binding cassette transporter A1
ACC: Acetyl-coA carboxylase
ALT: Serum aminotransferase
AST: Aspartate transaminase
AS-IV: Astragaloside IV
BSA: Bovine serum albumin
DNL: De novo Lipogenesis
FFA: Free fatty acid
FDA: Food and Drug Administration
GCN5: General control non-repressed protein 5
H3K9ac: Acetylation of histone H3 at lysine 9
H3K14ac: Acetylation of histone H3 at lysine 14
HATs: Histone acetyltransferases
HCC: Hepatocellular carcinoma
H&E: Hematoxylin and eosin
HFD: High-fat diet
KAT2A: Lysine acetyltransferase 2A
LDL-c: Low-density lipoprotein cholesterol
LW: Liver weight
LW/BW: Liver weight-to-body weight ratio
LXR: Liver X receptor
MASLD: Metabolic dysfunction-associated steatotic liver disease
OA: Oleic acid
PA: Palmitic acid
SREBP: Sterol-regulatory element binding protein
SREBP1c: Sterol regulatory element-binding protein 1c
SREs: Sterol regulatory elements
TC: Total cholesterol
TG: Triglyceride
THR-β: Thyroid hormone receptor-β
