# Supplementary material for: Astragaloside IV improves MASLD via GCN5-LXRα/SREBP1c signaling pathway-mediated hepatic de novo lipogenesis
Source: Chin Med. 2026 Sep 28;21:223. doi: 10.1186/s13020-026-01491-0 (PMC13617935; doi:10.1186/s13020-026-01491-0)
Supplement: Supplementary file 1 [file 13020_2026_1491_MOESM1_ESM.docx]

**Supplemental information**

**Figure S1. The effects of AS-IV on PA/OA-induced lipid accumulation in primary hepatocytes**

Primary hepatocytes were incubated PO (PA, 100 μM/OA, 200 μM) with AS-IV (50 μM) for 16 h. The treated cells were collected and used for subsequent analyses.

(A) The cellular TG contents were measured in the treated primary hepatocytes (n = 3).

(B) The cellular TC contents were measured in the treated primary hepatocytes (n = 3). Error bars are represented as mean ± SEM. Statistical analysis was done with one-way ANOVA (Dunnett’s post-test). **p* < 0.05, ***p* < 0.01 ****p* < 0.001.

**Figure S2. AS-IV treatment ameliorates HFD-induced hepatic steatosis via SREBP-1c-mediated DNL**

Primary hepatocytes were incubated PO (PA, 100 μM/OA, 200 μM) with AS-IV (50 μM) for 16 h. The treated cells were collected and used for subsequent analyses.

(A) Free fatty acid (FFA) uptake was measured in the primary hepatocytes (n = 4). (B) Oxygen consumption rate (OCR) was measured in the primary hepatocytes (n = 4). (C-D) The mRNA expression of lipid transportation and FA oxidation-related genes (n = 4). (E) The DNL was measured in the primary hepatocytes (n = 4). Error bars are represented as mean ± SEM. Statistical analysis was done with one-way ANOVA (Dunnett’s post-test). **p* < 0.05, ***p* < 0.01 ****p* < 0.001.

**Figure S3. Knockdown of SREBP-1c reversed the effect of AS-IV in hepatocytes**

Primary hepatocytes were transfected with the indicated plasmids for 24 h, then incubated PO (PA, 100 μM/OA, 200 μM) with AS-IV (50 μM) for 16 h. (A) The protein expression of SREBP1 was detected by western blot (n = 3). (B) The levels of SREBP1-targeted genes in cells (n = 3). (C-D) The cellular TG and TC contents were measured in the treated primary hepatocytes (n = 3). (E-F) The treated cells were fixed and stained with Nile Red and Bodipy (n = 5). Quantification of cellular cholesterol and neutral lipids was analyzed using Image-ProPlus. Error bars are represented as mean ± SEM. Statistical analysis was done with one-way ANOVA (Dunnett’s post-test). **p* < 0.05, ***p* < 0.01 ****p* < 0.001.

Figure. S1

Figure. S2

Figure. S3
